# Role of Soil Microbiota Enzymes in Soil Health and Activity Changes Depending on Climate Change and the Type of Soil Ecosystem

**DOI:** 10.3390/biology13020085

**Published:** 2024-01-29

**Authors:** Jokūbas Daunoras, Audrius Kačergius, Renata Gudiukaitė

**Affiliations:** 1 Life Sciences Center, Vilnius University, Sauletekis Av. 7, LT-10257 Vilnius, Lithuania; jokubas.daunoras@gmc.stud.vu.lt; 2 Lithuanian Research Centre for Agriculture and Forestry, Kedainiai Distr., LT-58344 Akademija, Lithuania; audrius.kacergius@lammc.lt

**Keywords:** microbial enzymes, climate change, soil ecosystems, carbon sequestration, soil enzyme activity, soil fertility, soil microbiota

## Abstract

**Simple Summary:**

Microorganisms and their enzymes are crucial to ensuring soil quality, health, and carbon sequestration. Their numerous reactions are essential for biogeochemical cycles. However, a comprehensive review is lacking to summarize the latest findings in agricultural and enzymatic research. Although the relationships between soil enzyme activities and different soil ecosystems, such as arctic and permafrost regions, tropics and subtropics, tundra, steppes, etc., have been intensively investigated, particularly in the context of climate changes, only a few reviews summarize the impact of climate change on soil enzyme activity. This review aims to highlight the main groups of microbial enzymes found in soil (such as α-glucosidases and β-glucosidases, phosphatases, ureases, N-acetyl-glucosaminidases, peptidases, etc.), their role in the global nutrient cycles of carbon, nitrogen, phosphorus, sulfur, carbon sequestration, and the influence of intensive agriculture on microbial enzymatic activity, and the variations in enzyme activity across different climate zones and soil ecosystems. Furthermore, the review will emphasize the importance of microbial enzymes for soil fertility and present both current challenges and future perspectives.

**Abstract:**

The extracellular enzymes secreted by soil microorganisms play a pivotal role in the decomposition of organic matter and the global cycles of carbon (C), phosphorus (P), and nitrogen (N), also serving as indicators of soil health and fertility. Current research is extensively analyzing these microbial populations and enzyme activities in diverse soil ecosystems and climatic regions, such as forests, grasslands, tropics, arctic regions and deserts. Climate change, global warming, and intensive agriculture are altering soil enzyme activities. Yet, few reviews have thoroughly explored the key enzymes required for soil fertility and the effects of abiotic factors on their functionality. A comprehensive review is thus essential to better understand the role of soil microbial enzymes in C, P, and N cycles, and their response to climate changes, soil ecosystems, organic farming, and fertilization. Studies indicate that the soil temperature, moisture, water content, pH, substrate availability, and average annual temperature and precipitation significantly impact enzyme activities. Additionally, climate change has shown ambiguous effects on these activities, causing both reductions and enhancements in enzyme catalytic functions.

## 1. Introduction

Soils accommodate a vast abundance of microorganisms, making up the majority fraction of the Earth’s global biomass. Among these microorganisms, bacteria dominate, accounting for 15% of the total living biomass, while fungi and archaea make up only 2% and 1%, respectively [1]. In contrast, animals represent only 0.3% of the total living biomass. [1,2]. The soil hosts the Earth’s most diverse and intricate microbiome, frequently containing over 0.5 mg of microbial biomass carbon (C) and exceeding 50,000 species per gram of soil [1,3,4]. More than 40 soil microbiome functions have been listed that either directly or indirectly impact soil, plant, animal, and human health [1].

The C source takes on various forms, such as polysaccharides (including cellulose, β(1-3) glucan, hemicellulose, chitin, and starch), which are broken down into dissolved organic C by enzymes like cellobiohydrolase (CBH), α-glucosidase (AG), β-1,4-glucosidase (BG), β(1-3) glucanase, xylanase (XYL), endo-chitinase, endo-cellulase, β-1,4-N-acetyl-glucosaminidase (NAG), or amylase [5,6]. The aromatic form of C, such as lignin, is affected by enzymes like phenoloxidases (PO) or peroxidases (PP) [5]. Aliphatic C compounds like fatty acid esters necessitate the action of lipolytic enzymes (LIP) [5]. The conversion of polymeric organic C into dissolved organic C by soil microbial enzymes stands as the critical, rate-limiting step in soil organic matter (SOM) degradation [7,8]. Microbial enzymes are pivotal in the turnover of soil C [9].

The nitrogen (N) sources can be classified based on their peptide and non-peptide nature. The peptide sources encompass proteins and peptides, requiring enzymes such as endo-peptidases, aminopeptidases, or carboxypeptidases for substrate hydrolysis [10,11,12]. Notably, the crucial enzymes responsible for N acquisition, such as leucine aminopeptidase (LAP) and NAG, stand out for their role in breaking down proteins and chitin [13,14,15]. On the other hand, the non-peptide sources include primary amines, influenced by various amidases and ureases (Ure) [5].

Inorganic nitrogen (N) primarily consists of nitrate and ammonium. Temperate climates often have higher concentrations of nitrate, whereas ammonium is more prevalent in many tropical soils [16]. The worldwide N cycle relies heavily on microorganisms that use nitrate as an alternative terminal electron acceptor. Nitrite produced during this process can be transformed into gaseous N compounds by denitrification or into NH_4_ via dissimilatory nitrate reduction to ammonia (DNRA). Denitrification and DNRA play a crucial role in soil nitrate reduction by reducing NO_3_^−^ and producing N_2_O—a potent greenhouse gas. These processes are influenced by the oxygen (O), N, C levels, moisture, pH, as well as the size and composition of the nitrate-reducing microbial population [17]. Nitrate reductase (NR) is a key enzyme in the N cycle that converts nitrate into nitrite [18]. Phosphorus (P) exists in the forms of diesters or monoesters, undergoing hydrolysis facilitated by phosphodiesterases, phosphomonoesterases, and phytases (PHY) [5]. To fulfill the P requirements, microbes secrete diverse phosphatases (acid (AcP) or alkaline AlP)) capable of hydrolyzing organic P, releasing an available inorganic form [13,19].

**Figure 1:**
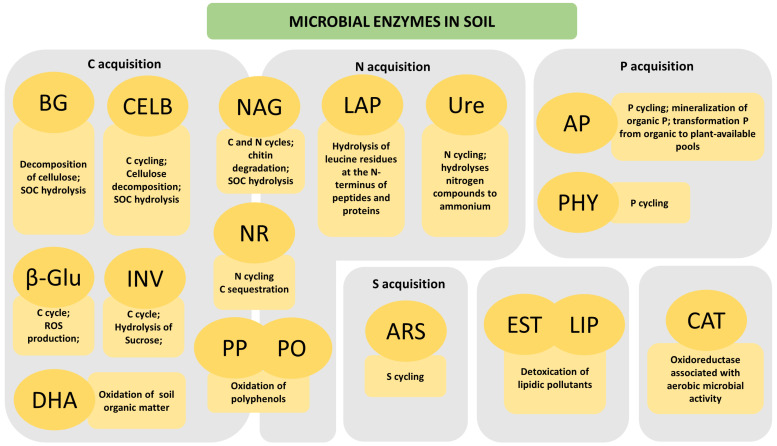
The main soil microbial enzymes and their roles in the soil. Acid/alkaline phosphatases (AP), arylsulphatase (ARS), β-1,4-glucosidase (BG), β(1-3) glucanase (β-Glu), catalases (CAT), cellobiohydrolase/exo- and endocellulases (CELB), dehydrogenases (DHA), esterases (EST), invertase (INV), leucine aminopeptidase (LAP), lipases (LIP), β-1,4-N-acetyl-glucosaminidase (NAG), nitrate reductase (NR), phenol oxidases (PO), peroxidases (PP), phytases (PHY), reactive oxygen species (ROS), soil organic carbon (SOC), ureases (Ure).

Fundamentally, AG, BG, NAG, AcP, AlP, Ure, and LAP represent the most frequently studied targets for analyzing soil enzyme activity. They act as the primary indicators for predicting soil health and fertility [7,20,21,22,23,24,25,26,27]. The main soil microbial enzymes and their roles in the soil are depicted in Figure 1.

Soil microbial activities, as demonstrated by the production of enzymes such as BG, NAG, LAP, and AP, reflect microorganisms’ metabolic and stoichiometric requirements [20]. These extracellular enzyme activities (EEA) connect ecological metabolic theory and ecological stoichiometry theory, which suggests a critical link between microbial metabolism and nutrient availability in the environment [28,29]. Additionally, the BG:NAG, BG:AP, and NAG:AP relative activities have been proposed as indicators of C vs. N, C vs. P, and N vs. P acquiring enzyme relative activities [20,21]. Recently, an increasing number of studies have used ecoenzymatic stoichiometry to assess the microbial nutrient limitations in diverse soils and evaluate the environmental impacts on soil health [27,30,31,32,33,34,35,36,37]. This method, based on the soil EEA, provides a practical and reproducible way to assess soil ecosystem status, revealing the effects of various environmental factors and management practices [38]. Because of their sensitivity, practicality, and association with soil biology and structure, soil enzymes, also known as the “biological fingerprints” of soil history, are regarded as valuable indicators of soil quality [38,39]. Nonetheless, the differences in EEA across soil ecosystems highlight the need for a comprehensive review of existing research.

The soil ecosystem is a crucial life-support system that comprises air, water, minerals, organic matter, and a rich diversity of macro- and micro-organisms. This intricate network is fundamental to terrestrial ecosystems, which are exclusively land-based. Among these, four main types are recognized, each of them associated with distinct geological zones: forest, grassland, tundra, and desert ecosystems [40].

Forest ecosystems are distinguished by deep-rooted trees, pronounced “litter layers” (O horizons), and the recycling of organic matter and nutrients by various soil-dwelling organisms. These characteristics set them apart from prairie, rangeland, and agricultural soils [41]. The health of forest soil is defined by its ability to function within ecosystem and land-use boundaries, maintaining plant and animal health, ecological biodiversity, productivity, and environmental quality [42].

Grasslands, which occupy approximately 25% of the terrestrial area, are crucial to the global C balance [43]. They include temperate and tropical (savanna) grasslands, dominated by grasses and herbs during the vegetation season. The role of enzymes in maintaining soil fertility in grassland ecosystems has been emphasized, with a specific focus on enzymes like BG, CAT, Ure, AP, cellulases, and invertase (INV) [44].

Tundra ecosystems, devoid of trees, are found in cold climates or areas with limited rainfall, such as the Arctic or mountain tops. Characterized by long winters and short summers, they predominantly feature Cryosol soils, formed in environments with underlying permafrost [45]. Permafrost is ground that remains at or below 0 °C for over two years [46].

Desert ecosystems are defined by minimal rainfall and sparse vegetation. They experience hot days and cold nights, with soils low in N and organic matter but high in CaCO_3_ and phosphate, contributing to their infertility [47].

Climate change and global warming have significant effects on soil ecosystems, particularly by altering the C storage and nutrient availability. The most pronounced impacts are anticipated in tropical and arctic regions. Deforestation, land abandonment, climate fluctuations, elevated CO_2_ levels, fertilization, and N deposition all have an impact on soil processes in the tropics. These effects differ based on the soil characteristics, nutritional status, and disturbance [48].

In the Arctic, rising temperatures are causing permafrost thaw, which accelerates microbial activity in tundra soils, releasing greenhouse gases and additionally contributing to climate change. This warming also promotes shrub encroachment in tundra zones, changing the number and quality of the plant inputs and affecting soil microbial activities [49,50]. Furthermore, as organic C in permafrost thaws, the Arctic tundra shifts from a net C sink to a net C supply due to increasing vulnerability to microbial decomposition [49].

Deserts, while being potentially large CO_2_ sinks, face challenges as rising soil temperatures can cause soil air expansion and increased CO_2_ release into the atmosphere under climate change circumstances [51]. A recent study has revealed differences in microbial community and biodiversity among soil environments [49,50,52,53,54,55,56]. The decline in soil microbial diversity also poses a significant danger to the ecosystem balance.

However, it is not just the diversity and abundance of microorganisms that matter but also their enzymatic activity in these soil types. Analyzing soil enzyme stoichiometry can provide insights into soil fertility, microbial activity, and the effects of global warming and climate change. There is growing interest in evaluating the microbial populations and enzyme activity in various soil ecosystems, emphasizing the need for a comprehensive review to better understand the current situation and summarize the findings.

This study aims to: (1) provide a comprehensive review of the most important microbial enzymes involved in soil fertility and health; (2) summarize the activities of microbial enzymes across various soil ecosystems; (3) analyze the effects of global warming and seasonal climate changes on enzyme activity, particularly in cold and tropical climate zones; (4) highlight the relationships between microbial enzyme activity and strategies for C sequestration; and (5) summarize the effects of fertilization or soil supplementation with other compounds, such as herbicides, on microbial enzyme activity. To the best of our knowledge, this study is the first to bring together the most significant contemporary aspects concerning soil microbial enzymes, including their diversity, activity, role in soils, and participation in the global C, N, P, S cycles, as well as the influence of climate change, soil ecosystems, and agricultural activities. However, it is important to note that the results obtained by researchers are highly variable and thus a unique system for the analysis of the same enzymes, climate factors such as temperature, soil moisture (SM), and precipitation, or a general data basis is required to evaluate the impact of abiotic factors like climate warming or fertilization more accurately.

## 2. Main Microbial Enzymes in Soil

The conversion of C, N, and P sources into soluble compounds is a major process involving different soil enzymes. However, other microbial enzymes, such as dehydrogenases (DHA), catalases (CAT), PO, LIP, and carboxylesterases (EST), are also important for soil quality and can be used as biological soil quality indicators [57,58,59]. In the next subsections, the most important microbial enzymes will be presented.

### 2.1. β-Glucosidases

BG is a common and predominant enzyme in soils and plays an important role in catalyzing the hydrolysis of various glucose derivatives present in the soil ecosystem. They are highly diverse enzymes owing to the wide diversity of glycosidic bonds in their substrates [60] Among all of the glycosidases, AG and BG, as well as α- and β-galactosidase, are the main members [38]. However, BG is more prominent in soil than AG and α- and β-galactosidases [38]. It is a rate-limiting enzyme in the microbial degradation of cellulose into glucose, an important C energy source of life for microorganisms in the soil [57]. The reaction performed by BG can be described using the following reaction (Equation (1)):

Glucoside + H_2_O = ROH + glucose (1)

BG enzymes are critical to the cellulose breakdown process. They specifically catalyze the terminal reaction by hydrolyzing cellobiose residues, and they also participate in the hydrolysis of maltose and cellobiose [38,60,61]. These processes, as described in Equation (1), eventually produce glucose as the end product [62]. BG activity is closely linked to soil organic matter (SOM), biological activity, and C cycling.

According to research, BG activity decreases significantly as the soil pH increases, particularly in paddy soil when the pH values move from 4.3/4.5 to 7.4/8.5 [57,63]. SM is also an important element in the C transformation activities mediated by BG [57]. Notably, BG activity has been shown to drop by 10–80% and 35–83% with a 10% and 21% reduction in SM, respectively, the magnitude of which varies with the soil depth [64]. Furthermore, an increase in soil salinity and solidity is associated with a considerable drop in BG activity, with both exponential and linear trends [65].

It was also shown that soils enriched with organic material with a high C:N ratio and high amounts of lignified roots have a lower BG activity and a slow organic matter decomposition [60]. Conversely, soils composed of easily decomposable organic matter tend to have increased BG activity. Therefore, adding soil organic residues, such as biosolids, manure, urban sludge, and poultry litter, increases the activity of this enzyme in the soil [60].

BGs are critical enzymes in soil, which primarily catalyze the hydrolysis of glucose derivatives, affecting cellulose degradation, C cycling and in such a manner ensuring the availability of C sources for the soil microbiota. However, their activity is strongly controlled by the soil pH, SM, and organic material content.

### 2.2. β-1,4-N-Acetyl-Glucosaminidases

There are three naturally occurring enzymes that degrade chitin: lytic polysaccharide monooxygenase (LPMO), chitinase, and NAG. LPMOs start the process by using an oxidative mechanism on the surface of crystalline chitin. They cause chain breakage and oxidized chain ends, which facilitates further breakdown by chitinases. Chitinases catalyze the breakage of glycosidic linkages in chitin chains, resulting in the production of chitin oligosaccharides and chitin dimers as the end products. NAG completes the degradation process by converting the chitinase products into N-acetylglucosamine, a monomer that can be used in a variety of metabolic processes [66].

In this way, NAG participates in chitin conversion into amino sugars, which are a major source of easily mineralizable C and N in soils [67,68]. The activity of NAG has been positively related to the soil organic C and total N. High inorganic N availability decreased NAG activity, while the soil pH was positively related to NAG activity [15]. NAG can be classified into four families of glycoside hydrolase based on the character of the amino acid sequence [69]. Depending on the family, they can function at acidic, neutral or slightly alkaline pHs [69].

In summary, NAG has an important function in the breakdown of chitin and related polymers and is essential for turning chitin into mineralizable C and N, with the activity affected by soil organic C, total N, inorganic N availability, and soil pH.

### 2.3. Invertases

INV, also known as β-fructosidase, β-fructofuranosidase, β-d-fructofuranoside fructohydrolase, sucrase, invertin, saccharase, and other names [70], is a carbohydrase that catalyzes the hydrolysis of sucrose to yield D-glucose and D-fructose in equimolar proportion, known as invert sugar [71,72]. This enzyme, which is related with the C cycle, indicates soil microbial activity and the intensity of C metabolism [73]. INV hydrolyzes the α-1,2-glycosidic link in sucrose in the surrounding milieu and is efficient at distinct pH ranges, thus classed as acidic (pH 4.5–5.5), neutral, or alkaline (pH 6.5–8.0) [72]. Bacterial INVs can be classified as intracellular/endo or extracellular/exo enzymes. They have a high selectivity for sucrose; however, some studies have revealed that they have about 10% activity toward other disaccharides such as raffinose, maltose, trehalose, lactose, melibiose, and cellobiose [72]. According to research, the addition of organic matter stimulates soil INV activity, which is required for organic matter decomposition and humus synthesis [74].

### 2.4. Leucine Aminopeptidases

Aminopeptidases are proteolytic enzymes that selectively hydrolyze single amino acids or dipeptides at proteins’ N-termini [75]. LAPs are metallopeptidases from the M17 family that catalyze the hydrolysis of Leu residues at the N-terminus of proteins and peptides [75,76]. However, most members of the M17 family have a wide substrate range that frequently includes amino acids, such as Met, Ala, Arg, and Ile, in addition to Leu [75].

LAP enzymatic activity can be dependent on a variety of circumstances. LAPs typically work best at pH 8–9, and their activity in the presence of divalent metal ions varies by strain. Optimal enzymatic activity is often reported with Mn^2+^ as a cofactor, but activity is also shown with Ni^2+^, Co^2+^, and to a lesser extent, with other metals [75].

LAP is a housekeeping protease, one of numerous enzymes found in soils that help bacteria absorb N [20,77]. LAP activity has been shown to decrease when the inorganic N supply is high [15]. LAP is also susceptible to cadmium (Cd), as demonstrated by researchers who discovered that the Cd inhibition of LAP activity increased as the Cd concentrations increased and that Cd exhibited noncompetitive inhibition on LAP [78]. The addition of clay minerals was found to lower the LAP activity and maximum reaction rate (V_max_). This suppression by Cd could be attributed to the displacement of metals involved in the enzyme structure and the occupation of the enzyme’s active core [78].

LAP is crucial among the microbial enzymes in soils, used in measuring soil enzyme stoichiometry, and is helpful in predicting soil fertility and the impact of different compounds on soil health.

### 2.5. Ureases

Soil ecosystems rely heavily on urea amidohydrolase—Ure for their N cycle. It drives urea’s (NH_2_)_2_CO) hydrolysis, which releases ammonium ions (NH_4_^+^) and bicarbonate as an intermediary. This raises the pH of the soil and causes N to be lost to the atmosphere via NH_3_ volatilization [57,79,80]. The primary reaction carried out by Ure is shown in Equation (2).

Urea = 2 NH_3_ + CO_2_ (2)

Because of its role in the microbially induced precipitation of calcium carbonate (MICP), Ure has attracted a lot of attention. Ure activity-based MICP is advantageous for soil healing, slope stabilization, settlement reduction, erosion control, and liquefaction prevention [81,82,83]. For example, autogenous *Staphylococcus* can serve as a microorganism to reinforce the structure of the soil crust [84]. The process of mineralizing desert soil requires a pollution-free microbial treatment technique that is easy to implement in desert conditions. It was observed that the strength of the soil increased when the Ure activity was in the range of 4.4–9.5 mM hydrolyzed urea per minute, indicating that maintaining a level of Ure activity is crucial for successful biocementation [85]. It was also proposed that from soil isolated Ure-positive *Staphylococcus* sp. H6 can successfully reduce the water permeability and improve the soil quality [86].

Furthermore, Ure activity influences the rate of nitrogenous nutrient introduction into the soil and can be used as an indicator to determine the gain or loss of N [87,88]. Since its activity increases with organic fertilization and decreases with soil tillage, it has been frequently used to assess changes in soil quality caused by management [38]. Several factors affect Ure stability, including the temperature, moisture content, microbial community, and physical and chemical characteristics of the soil [89]. Ure also catalyzes the hydrolysis of hydroxyurea and semicarbazide and contains Ni^2+^ ions as a co-factor [38,57,88].

Ure is essential in soil ecosystems for its role in the N cycle, particularly in hydrolyzing urea to release ammonium ions and influencing the soil pH. Ure activity, varying with factors like organic fertilization, soil tillage, and environmental conditions, is a crucial indicator of soil quality and N management in agricultural and ecological contexts.

### 2.6. Acid/Alkaline Phosphatases

Soil APs play an essential role in the mineralization of organic P, especially in tropical regions [90,91]. APs are enzymes that catalyze the hydrolysis of phosphomonoesters and, in some cases, phosphodiesters, resulting in the release of phosphate (Equation (3)).

Phosphate ester + H_2_O = ROH + PO_4_^−^ (3)

APs also can hydrolyze phosphoric acid anhydrides [20,57]. Because plants only use inorganic P and a large portion of soil P is organically bound, mineralization of this organic portion can have a significant impact on plant nutrition [92]. P transformation-related AP hydrolyzes the ester bonds in organic P in soil to promote P conversion, thus impacting plant P availability [93]. When there is a lack of P in the soil, plant roots and microorganisms increase AP secretion in order to boost phosphate solubilization and remobilization, influencing the plant’s ability to endure P-stressed conditions [94]. Ps are typically activated when the soil P availability is low.

Phosphomonoesterase is the most extensively studied soil AP, and it plays a crucial role in the biogeochemical cycling of P. It catalyzes the hydrolysis of phosphate monoesters, resulting in the production of free phosphate, which is essential for biological uptake [57,95,96]. This enzyme targets low molecular weight P compounds with monoester bonds, including nucleotides, sugar phosphates, and polyphosphates. Its activity is contingent upon the soil’s pH, functioning under both acidic and alkaline conditions, depending on its optimal pH [97]. Consequently, acid phosphatase (AcP) activity is more prevalent in acidic soils, while alkaline phosphatase (AlP) activity predominates in alkaline soils. The soil pH significantly influences the rate of AP synthesis, its release, and stability [98].

AP activity was influenced in soils affected by forest fire, increasing over time as the soil recovered [57]. Drought has also been proposed as an influence—when the SM was reduced by 21%, AcP activity was reduced by 31–40% [64]. It was also discovered that APs can be inhibited in organically amended soils, whereas mineral fertilization increased this enzyme activity. It was also proposed that lead and other heavy metals in the soil reduced AP activity [99].

AP is key to converting organic P into a form usable by plants, with its activity influenced by the soil pH, environmental stressors, and heavy metals. This enzyme’s functionality directly impacts plant growth and soil health, adapting to varying environmental conditions.

### 2.7. Sulfatases

The majority of soil sulfur (S) exists as organic S, accounting for 90 –98% of the total S. Sulfate ester accounts for 30–75% of the organic S in soil [100]. It was suggested that the sulfate ester pool is the most important organic S for soil microorganisms [101]. Thus, the mineralization of organic sulfates is an important step in increasing soil S availability [102]. Arylsulfatases (ARS) are enzymes found throughout nature that catalyze the release of SO_4_^2−^ from sulfate esters. Equation (4) depicts the reaction principle of ARS.

ROSO_3_^−^ + H_2_O = ROH + SO_4_^2−^ (4)

ARS is an indicator of S mineralization in soil and plays an important role in S cycling [68,102]. Bacteria secrete them into the environment in response to S limitation [101,103]. Researchers have discovered a significant negative relationship between the ratio of ARS to BG activities and the concentrations of soluble and adsorbed sulfate in soils. This finding indicates that microorganisms tend to produce more ARS when the concentration of sulfate is low in the soil. Additionally, this suggests that the production of ARS by soil microorganisms is likely a response mechanism to adapt to lower sulfate availability [101]. ARS activity in different soil systems is often correlated with the microbial biomass and rate of S immobilization. This enzyme has also a role in the hydrolysis of aromatic sulphate esters (R–O–SO_3_) into phenols (R–OH) and sulfate [104].

ARSs, as indicated by their negative relationship with soluble and adsorbed sulfate concentration, are essential in regulating the S availability in soils, particularly under conditions of S scarcity. This enzyme’s activity, which varies across different soil systems, is closely linked to the microbial biomass and S immobilization, and it highlights its critical role in both soil health and the broader environmental S cycle.

### 2.8. Dehydrogenases

The activity of DHA is commonly used as an indicator of biological activity in soils [22,105]. DHA is an intracellular enzyme that belongs to the oxidoreductases family. DHA is known to oxidize SOM by transferring protons and electrons from acceptors to substrates. Equation (5) shows the main equation for DHA activity. These processes are part of the soil microorganism respiration pathways, and DHA activity is strongly dependent on the metabolic state of the soil biota and is significantly correlated with the soil biomass C [90].

XH_2_ + A = X + AH_2_ (5)

After nutrient adjustments, DHA activities rise with increasing microbial populations [105,106]. Because these processes are part of the respiration pathways of soil microorganisms, research on the activity of the DHA enzyme in soil is critical because it can indicate the soil’s ability to support biochemical processes that are essential for maintaining soil fertility and health.

### 2.9. Other Enzymes

Other microbial enzymes, such as cellulases, LIP, CAT, PP, PO, NR, are less often introduces in the measurements of soil EEA. This is largely due to their specific roles in soil processes, which are less directly linked to the primary C:N:P cycles that are traditionally emphasized in soil health assessments. However, they still play a significant role in ensuring soil fertility and could be introduced into EEA models to predict soil microbial activity more precisely.

#### 2.9.1. Cellulases

Cellulases are enzymes that catalyze the hydrolysis of cellulose into D-glucose. Cellulose is the most abundant structural polysaccharide in plant cell walls, with β-1,4-glucosidic linkages, accounting for nearly 50% of the biomass synthesized via photosynthetic CO_2_ fixation [38]. Three key enzymes are involved in the degradation of SOM from cellulose to glucose: endoglucanase (endo-1,4-D-glucanase), CBH, and the aforementioned BG [107]. Endoglucanase randomly cleaves β-bonds within the cellulose molecule, whereas CBH removes cellobiose units from cellulose chain ends [107]. Additionally, the temperature, soil pH, water and oxygen contents, the chemical structure of organic matter and its location in the soil profile horizon, the quality of organic matter/plant debris and soil mineral elements, a trace elements from fungicides, all influence cellulase activity in agricultural soils [38]. Cellulases are essential for converting cellulose into glucose, and they are key players in the decomposition of SOM. Their activity, influenced by a range of environmental factors like the temperature, soil pH, and mineral content, reflects the delicate balance of soil ecosystems and the complexity of nutrient cycling in agricultural settings.

#### 2.9.2. Lipolytic Enzymes (Lipases, Carboxylesterases and Other Esterases)

LIP can be used to evaluate the decontamination treatment of oil-polluted soils and to detoxify harmful toxic substances in soil. The induction of soil LIP activity is a useful indicator of oil biodegradation in naturally attenuated (unfertilized) and bioremediated (fertilized) soils, allowing for a quick and accurate assessment of decontamination treatment following an oil spill [108]. LIP enzymes are important in the removal of pollutants from environmental matrices.

Phosphate esters (PAEs) are one of the most interesting pollutants [58]. PAEs are often used in adhesives, pesticides, and cosmetics as additives [109,110]. However, they are easily released from products and migrate into various environmental matrices, such as water, air, soil, and sediments, because they are not chemically bound to plastic materials [111,112]. PAEs have been discovered in terrestrial, aquatic, and indoor environments [113,114]. They were then converted into monoalkyl phthalate esters and phthalic acid by EST and LIP, which significantly decreased the half-lives of the PAEs [58].

Pyrethroids are a class of synthetic organic insecticides extensively used in agriculture and households for pest control and disease transmission prevention. Due to this substantial usage, they have become a notable source of environmental pollution. This has led to concerns about food safety and human health [115]. However, microorganisms capable of degrading pyrethroids, along with relevant LIP/EST enzymes, have shown efficient abilities in breaking down these compounds. They do this primarily by hydrolyzing the ester linkage in pyrethroids, leading to the formation of carboxylic acids and alcohols. These substances are further metabolized into several key metabolites, including 3-phenoxybenzoic acid (3-PBA), 3-phenoxybenzaldehyde (PBAld), 3-phenoxybenzyl alcohol (PBAlc), and 2-(4-chlorophenyl)-3-methylbutyric acid (CLAc). These metabolites are commonly observed in the microbial elimination of pyrethroids [115,116].

LIP enzymes are vital for assessing soil decontamination, particularly in oil-polluted soils, and play a crucial role in detoxifying harmful substances, indicating their effectiveness in environmental remediation and their impact on soil fertility. Additionally, it is important to include LIP in quantitative models while researching soil fertility and soil health.

#### 2.9.3. Phenol Oxidases, Peroxidases

PO and PP are expressed for a variety of reasons, including ontogeny, defense against pests and pathogens, and C and N acquisition [117]. In the presence of oxygen, POs are known to play an important role in the breakdown of polyphenol compounds (lignin, tannin, and their degradation products), whereas PPs use hydrogen peroxide (H_2_O_2_). When phenolic hydrogen or hydrogens are removed from polyphenols, radicals or quinones are formed [118]. Equation (6) depicts the main reaction.

A + H_2_O_2_ = oxidized A + H_2_O (6)

PO and PP have lower environmental stability than extracellular hydrolases, especially when associated with organic particles. Interaction with mineral surfaces has an impact on their activities, both positively and negatively. Across ecosystems, PO and PP activities generally increase with the soil pH [117]. PO and PP are key in breaking down complex polyphenols in soils, with their activity influenced by the environmental conditions and soil pH. These enzymes are valuable indicators of soil quality, reflecting the dynamic nature of soil biochemical processes.

#### 2.9.4. Catalases

CATs are intracellular enzymes involved in microbial aerobic activity and strongly influenced by the metabolic state of the soil biota [90]. CAT degrades H_2_O_2_ into O_2_ and H_2_O, protecting cells from reactive oxygen species [119]. It has been proposed that microbial communities that are subjected to higher levels of native oxidative stress have higher basal intracellular CAT concentrations and specific activities per biomass. Furthermore, high biomass soils typically have high CAT-specific activities per gram soil [120]. CATs are organized into three major classes comprising monofunctional enzymes containing either Fe-heme or binuclear manganese (Mn_2_) metal cofactors, and bifunctional catalase-peroxidases containing Fe-heme cofactors [120]. CAT also plays a pivotal role in various biotechnological applications. It is particularly important in bioremediation, where it serves as an indicator of hydrocarbon degradation in soil. This function is crucial to the bioremediation of crude oil pollution. CAT is also involved in providing oxygen in aerobic bioremediation processes. Beyond environmental applications, CAT has practical uses in the industrial sector, such as in the removal of hydrogen peroxide (H_2_O_2_) from bleaching industry effluents. Additionally, its properties make it a potential candidate for use as a food additive [121].

CAT serves a vital function in degrading H_2_O_2_ to protect cells from oxidative damage, which is crucial in microbial aerobic activities in soil. Its diverse applications, from the bioremediation of hydrocarbon pollution to industrial uses like effluent treatment, highlight the enzyme’s versatility and importance in both environmental and industrial biotechnology.

#### 2.9.5. Nitrate Reductases

Soil NRs are not often used to assess soil enzyme stoichiometry, but they play an important part in the N cycle and can be utilized to analyze and evaluate soil fertility. Inorganic N in soil consists mostly of nitrate (NO_3_^−^) and ammonium (NH_4_^+^), with NO_3_^−^ being more prevalent in temperate climes and NH_4_^+^ in tropical soils [16]. NO_3_^−^ reduction is one of the most essential steps in N recycling in nature, and it performs multiple functions: (1) NO_3_^−^ assimilation serves as a source of N; (2) as a terminal electron acceptor, it generates metabolic energy via NO_3_^−^ utilization (nitrate respiration); and (3) to keep the oxidation–reduction balance by discarding surplus energy (nitrate dissimilation) [16]. Additionally, the pH influences denitrification via enzyme sensitivity [17], with a pH of 7.0 to 8.0 being ideal for denitrification. Relationships between NR activity, denitrification, and C availability in soil have also been suggested [17]. The C substrate degradation pathways in the TCA cycle generate NADH, which provides electrons to denitrifying enzymes. The presence of labile organic C substrates in soils can boost denitrification rates and decrease N_2_O:N_2_ ratios in C-limited soils, lowering greenhouse gas emissions [17].

Soil NRs are essential in the N cycle, impacting soil fertility and functioning as a functional marker for assessing soil health [122]. Their role in nitrate assimilation, respiration, and the oxidation–reduction balance, coupled with their sensitivity to the soil pH and C availability, underscores their significance in nutrient cycling and greenhouse gas emission reduction.

In summary, the enzymes discussed in Section 2 have a significant impact on soil health and fertility. They function as catalysts in several metabolic processes, including nutrient cycling and the breakdown of SOM. Gaining a comprehensive understanding of the mechanisms and interactions of these enzymes is crucial to improving soil productivity and sustainability across many ecosystems. Acquiring this knowledge is essential for developing effective techniques to manage soil health, particularly considering evolving climate conditions and agricultural practices.

## 3. Soil Microbial Enzyme Activities and Challenges in Different Soil Ecosystems

The rate of soil microbial metabolism and biochemical cycling processes is reflected in soil enzyme activity. Soil enzymes decompose complex organic matter and convert it into plant-available nutrients, and this is an important factor in determining SOM decomposition and nutrient cycling [20,123,124]. Enzyme stoichiometry (e.g., βG:(NAG + LAP)) reflects microbial assemblage nutrient requirements and environmental nutrient availability [20]. The soil depth, woodland, pH, temperature, and vegetation type all have a significant impact on soil enzyme activity in various soil ecosystems [125,126].

### 3.1. Forest and Grassland

Forests and woodlands cover roughly one-third of the Earth’s surface and play important roles in global C sequestration and nutrient cycling. Forests, in comparison to other terrestrial plant communities, are frequently highly heterogeneous environments [127]. The SOC and soil total N were found to be important in influencing EEAs in both forest and grassland soils [20,23,128,129,130]. Researchers also revealed that organic matter greatly impacted enzyme activity in both forests and grasslands, while the pH and humic compounds affected forests only, and the humic compound mass and Ca content affected grasslands [23]. The crucial factors were the organic matter content, pH, and occasionally C/H ratio. Most enzymes were more active in grasslands, notably BG, CBH, phosphodiesterase, and alanine aminopeptidase. BG and CBH increased with the soil pH, while XYL, endoxylanase, and AP decreased. The soil humic content lowered BG but increased XYL. Enzyme activities were generally lower in tilled fields, especially Mn-peroxidase and ARS, which were significantly higher in grasslands [23]. The SM affected BG, CBH, and NAG activities seasonally [131]. In central China, afforested lands showed higher investment in C-hydrolyzing enzymes than N-hydrolyzing enzymes, alleviating N limitation after afforestation [132]. Woodlands had a lower enzyme C:N ratio than shrublands, possibly due to litter input and higher N fixation in shrublands [132]. It can be surmised that landscaping, biodiversity of plants, humus content, and litter play a significant role in microbial enzyme activity in forest and grassland.

### 3.2. Tropical and Subtropical Regions

Tropical ecosystems, which contain a significant portion of the global soil organic C stock and have high rates of primary production and respiration, are very important to the global C cycle [21]. Many tropical soils face base cation and P limitations as they age and weather, which affects primary production and organic matter breakdown [130,133,134,135,136]. On the other hand, N availability tends to be high, particularly in lowland forested areas, due to the abundant fixation rates and favorable environmental conditions [21]. Tropical soils have lower BG:AP and NAG:AP ratios than temperate ecosystems, especially in older or acidic soils, showing a higher demand for P relative to its availability [20,21]. Climate factors such as the temperature and precipitation show correlations with enzyme activities in tropical soils, indicating that microbial enzyme allocation may be regulated. The low microbial growth efficiencies in P-limited soils suggest that P availability influences C cycling in highly weathered tropical soils [21].

It was found that subtropical steppe soil microorganisms faced limitations in P, C, and N [27]. The enzyme C:N ratio correlated with the mean annual precipitation (MAP), mean annual temperature (MAT), clay content, soil C:N ratio, microbial biomass, and litter [130,132,137,138]. Additionally, it was noted that the lower BG:AP ratios in tropical soils versus higher latitudes mirror plant foliage elemental patterns [20]. N limits young soil productivity, while P constrains older soils due to weathering and leaching, potentially prompting higher microbial investment in enzymes targeting organic P over C or N [21]. Researchers suggested lower C:N and C:P ratios in enzyme activity in a temperate grassland compared to a tropical forest soil [137]. This illustrates how nutrient limitations, particularly P and N, as well as climate factors, impact microbial enzyme allocation and C cycling in tropical and subtropical ecosystems, affecting soil nutrient availability and ecosystem functioning in various environments.

### 3.3. Arid Lands/Desert

Arid lands, with annual precipitation of less than 500 mm, cover more than a third of the Earth’s landmass, which forms the largest terrestrial biome [26,139]. Deserts are ecologically defined by their vegetation, which consists of xerophytic plants such as shrubs and sparse woody vegetation, due to water scarcity [140]. Both environments have limited water resources, which slows organic matter decomposition when compared to more humid regions. As a result, desert ecosystems are regarded as stressful environments, with little available energy and nutrients for soil microorganisms [141].

Various desert types in western China were studied, measuring soil microorganism activities using specific enzymes related to C, N, and P acquisition. The combined enzyme activity ratios in deserts were found to be close to the global average (1:1.1:0.9), indicating that enzymatic stoichiometry is similar around the world [26,142]. However, the researchers proposed that soil C and N limit microbial metabolism. The microbial N limitation increased across desert types, from gravel to sand, mud, and salt deserts [26].

A study of desert steppes demonstrated differences in the enzyme activity ratios. The aforementioned desert steppes possessed higher C:N ratios than meadows and typical steppes, which indicates that the latter two had more N limitation. Meadow steppes had the highest C:P and N:P ratios, suggesting that P was scarcer in typical and desert steppes. Furthermore, as the soil depth increased, so did investment in N- and P-acquiring enzymes [137].

### 3.4. Saline Regions

Soil is a diverse and intricate ecosystem that is often exposed to multiple stressors simultaneously, either caused by human activities or natural causes [143,144]. For example, salinity is a major stress factor that increases the availability and toxicity of soil heavy metals like Cd and others. The combination of increasing salinity and heavy metal content results in a decrease in the rate of soil microbial respiration, microbial biomass, and enzyme activity. These changes have significant consequences for the health of the soil and the cycling of nutrients.

Enzymes such as AP and ARS play a crucial role in the cycling of P and S. These enzymes have reduced activity in saline environments, especially in soils contaminated with Cd. This decreased activity indicates a reduction in the availability of both P and S in these soils [145]. Furthermore, it has been observed that the harmfulness of lead (Pb) [146] and Cd [147] to the activity of Ure is also enhanced by salinity.

Soil salinity and metal pollution are prominent variables in dry and semi-arid regions, where soil degradation is a serious concern. The combined impact of these stressors often leads to synergistic interactions in arid soils. In such environments, the coexistence of multiple stressors is the norm rather than the exception [144]. This recognition emphasizes the necessity of implementing an effective strategy for soil management and remediation in these vulnerable regions.

Because of the many changing variables in different soil ecosystems, they have a significant impact on microorganisms and their enzymatic activity. The most important findings related to enzyme activities in the aforementioned soil ecosystems are presented in Table 1.

The examples presented in Table 1 suggest that the activities of key soil enzymes, including BG, Ure, AP, and LAP, vary based on factors such as the forestation level, temperature, SOM, and soil depth. A lower SOC content was associated with reduced BG activity. Additionally, as the soil depth increased, the activities of C-, N-, and P-acquiring enzymes decreased. However, drawing definitive conclusions about the activities of BG, LAP, NAG, and AP in different soil ecosystems is challenging and requires further research. Furthermore, developing a unified system to evaluate the activity of these enzymes could more accurately predict differences in various soil ecosystems.

## 4. Role of Microbial Enzymes in C Sequestration and Enzyme Activity Shift through Climate Changes

### 4.1. Enzymes Activity at Different Climate Conditions

According to the Intergovernmental Panel on Climate Change [154], if current greenhouse gas emissions continue, there will be a projected increase of 2.1–3.5 °C of global temperature by the years 2081–2100. C plays a crucial role in the complex interaction that influences soil fertility and climate patterns [155]. C sequestration is a crucial process that involves capturing and storing atmospheric CO_2_, which helps in reducing its concentration in the atmosphere and thus mitigates the negative impacts of climate change. The C cycle involves the storage of approximately 50% of the substances produced from photosynthesis in biomass and SOM, while the remaining portion is released into the atmosphere as CO_2_ through the plant or microbial respiration and SOM decomposition [155]. Soil microorganisms have a significant impact on the C budgets of ecosystems. They play various roles, such as decomposers, plant symbionts, or pathogens, which affect the availability of nutrients and the turnover and retention of C in the soil [3]. The relationship between different enzyme activities emphasizes the ability of soil microbes to serve as a reliable indicator of soil functionality. This presents a promising opportunity to obtain accurate information that can be used in ecosystem modeling as well as creating strategies for conservation and management in response to global change [156,157].

Changes in climate, especially in the SM, temperature, and CO_2_ levels, can change the activity of microbes in the soil [158,159,160,161]. Additionally, some soil ecosystems are more sensitive to these changes than others. For example, Arctic or desert ecosystems are more vulnerable than others [162]. Researchers have also found that as temperatures rose, the diversity of bacteria in Arctic soils became much less even [163]. Similar research was conducted in Oklahoma prairie soil, which revealed that warming temperatures increased the number of microbes by 40–150% while lowering diversity and greatly changing the make-up of the microbe community [164]. During droughts, the warming effect caused the soil to lose a lot of water, which made it difficult for plants and microbes to grow, leading to a 50–80% drop in their numbers [164]. As a result of overgrazing and climate change, global problems like grassland degradation have been reported. Studies have shown that C-acquired enzymes are more sensitive to warming than N-acquired enzymes [6,7]. The grasslands in northern China, which are an important part of the landscape of Eurasia, are about to change because of greater precipitation in the summer and increased N deposition. These huge global change agents are mostly controlled by enzyme activities in the soil, which drastically change the grassland ecosystem [93]. Climate changes that only last a short time can affect the C and P needs of microbes without necessarily changing the structure of the microbe community [165].

Forest soils are important C reservoirs that are intricately linked to terrestrial C cycling processes. In particular, global warming promotes the decomposition of SOC, increasing the C flux from the soil to the atmosphere [166]. This temperature increase may cause a decrease in microbial biomass, especially if labile C pools are depleted, limiting the microbial impact on C degradation [167]. An investigation was performed by looking into the effects of 1 °C warming on soil microbial communities and enzyme activities across different soil aggregate sizes, and it found that while certain enzymatic activities remained unaffected, such as BG, CBH, and NAG activities, warming caused a significant decrease in AcAP activity while increasing oxidase activities [168]. These findings indicated a higher sensitivity of the soil microbial community composition to warming effects within larger macroaggregates [168].

The soil enzymatic stoichiometry of C:N:P tends to align at a balanced ratio of 1:1:1 on a global scale [20]. However, global changes can significantly alter this equilibrium. A link was discovered between the N:P enzyme ratio and 19 years of experimental warming in tundra regions, which corresponded to changes in the mineral N and P pools during the growing season [169]. This shift in soil nutrient availability was accompanied by increased microbial EEA to degrade N-containing organic compounds and decreased activity to degrade P-containing organic compounds [169]. Climate change has been proposed to increase soil enzyme activity, thereby accelerating nutrient mineralization processes [170,171]. A positive relationship between Ure activity and temperature in grassland soils was discovered while exposed to temperatures ranging from −2 to 21 °C [172]. Furthermore, increased precipitation has the potential to increase EEA in soil via increasing enzyme and substrate diffusion [20,173,174].

**Figure 2:**
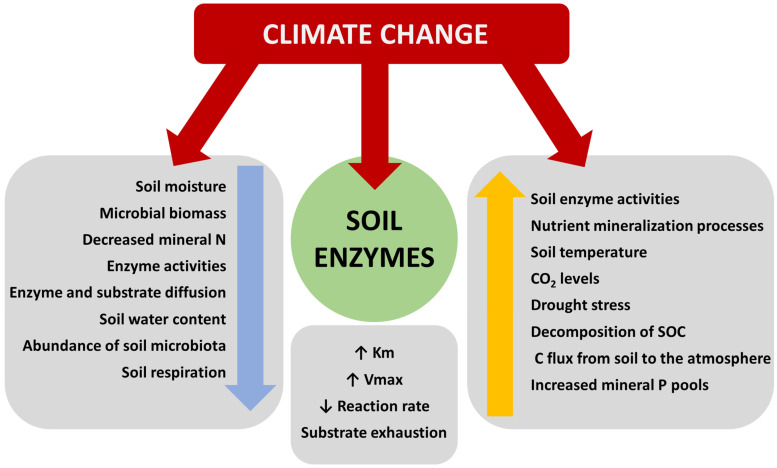
The diagram depicts how climate change impacts soil enzymes, detailing both inhibitory and stimulatory effects. SOC—soil organic carbon. Blue arrow indicated the properties that are decreased; yellow arrow indicated the properties that are increased.

Notably, researchers demonstrated that warming and increased precipitation had variable effects on different enzymes across soil depths, affecting activities such as AcAP and NAG in surface and subsurface soils differently [174]. The interaction of warming and increased precipitation had a significant impact on certain enzymatic activities, highlighting the complexities of these relationships [174]. Figure 2 illustrates the various effects of climate change on the environment, soil, and enzyme activities.

Climate change can reduce the SM and microbial activity, reducing enzyme activity, while also potentially increasing the soil temperature and CO_2_ levels, stimulating enzyme activity and nutrient mineralization. It is important to point out the contradictory nature of these findings, highlighting the strong reliance of effects on specific soil ecosystems.

#### 4.1.1. Arctic and Permafrost Regions

Global warming poses a significant threat to high latitude and cold ecosystems, which are important components of the Earth’s environment [175]. Arctic soils are experiencing accelerated organic matter decomposition and subsequent greenhouse gas emissions as a result of warming trends, reinforcing the climate change cycle [176,177,178]. Temperature increases are expected to have an impact on enzyme activities in these soils due to changes in the SM, potentially limiting microbial activity and C degradation [176,179,180].

Additionally, if enzymes from colder environments are locally adapted, the impact of rising temperatures on these enzymes may be reduced. Cold-adapted microorganisms’ enzymes are fine-tuned to function optimally at lower temperatures by lowering reaction activation energy (Ea). On the other hand, enzymes adapted for higher temperatures have less strict requirements to minimize Ea due to their inherent kinetic energy [181]. Lower Ea results in a temperature-independent reaction, which shows the potential for maintaining decomposition processes at lower temperatures through developing microbial enzymes that operate in spite of temperature changes [182]. Furthermore, climate-induced changes in enzyme systems may result in the expression of different sets of isoenzymes [175]. In response to warming, microorganisms may produce isoenzymes optimized for higher temperatures, which allows them to adapt to environmental changes [183].

Global warming causes rapid and profound changes in permafrost regions, resulting in collapsed ground features and significant changes in soil properties such as moisture, pH, C and N content, which impact the soil C cycle [152]. Changes in permafrost caused by warming have been observed to increase primary production and stimulate microbial activity [184]. The Qinghai–Tibetan Plateau, known as the “third pole of the Earth,” has been identified as the world’s largest low-latitude permafrost region, encompassing over 70% of the total alpine permafrost area in the Northern Hemisphere. This region is considered more vulnerable to climate change than the Arctic due to its high altitude and cryospheric environment [185]. Surface air temperatures in this region have risen at about twice the global rate (0.2 °C per decade) over the last five decades. This warming has caused widespread permafrost degradation in both high-altitude and high-latitude areas [152,186]. Permafrost degradation can make previously preserved C more accessible to microbial decomposition, increasing the soil respiration rates and releasing significant amounts of greenhouse gases, reinforcing the cycle of climate warming in a positive feedback loop [152,187,188].

Field experiments in alpine meadows revealed that five years of experimental warming stimulated soil microbial activity. This resulted in increased EEA activities, such as AcAP, INV, and Ure. However, the soil CAT activity decreased as a result of the reduced SM caused by warming [124]. On the other hand, it was also discovered that while both short-term and long-term warming altered the soil N cycling and increased Ure activity, it had no effect on the activities of soil cellulase, CAT, and AP in alpine meadows [189].

A recent study investigating the effects of intensive warming on soil enzyme properties in cold-adapted alpine grasslands in the Tibetan Plateau, conducted at temperature thresholds (around 20–25 °C), showed sudden reductions in substrate affinity, which in turn resulted in decreased temperature sensitivity and catalytic efficiency [175]. Even above 25 °C, enzymes critical for decomposing recalcitrant C compounds remained temperature-sensitive, potentially explaining the accelerated decomposition of such compounds.

#### 4.1.2. Tundra and Boreal Systems

It was proposed that N availability is a significant constraint in Arctic tundra ecosystems. Given that N is a critical component in the composition of microbial communities and the synthesis of extracellular enzymes, it has been proposed that the limited availability of N for soil microorganisms, which is required for their growth and enzymatic processes, limits the decomposition rates of SOM in tundra regions [153]. In one study, a 0.5 °C increase in the soil temperature was accompanied by a 22% decrease in the soil water content, resulting in a 50% decrease in the abundance of bacterial and fungal populations. In addition, the activity of the chitin-degrading enzyme NAG decreased noticeably. Although the soil respiration decreased by up to 50%, this was mostly seen late in the growing season [190]. These changes coincided with significant changes in the structure of the active fungi community. In response to warming, the relative abundance of a dominant *Tomentelloid* fungi decreased, while the relative abundance of *Ascomycetes* and *Zygomycetes* increased. Furthermore, a slight increase in soil ammonium and nitrate availability was observed. Temperature increases in northern-latitude ecosystems may not always result in a positive feedback loop in the soil C cycle, particularly in boreal forests with drier soils [190].

#### 4.1.3. Tropics and Subtropics

In tropical and subtropical regions, only a few field experiments have been conducted to investigate the impact of warming on microbial communities, enzyme activities, and SOC decomposition [191]. In a study carried out in a subtropical plantation in southeastern China, warming was found to increase the activity of the C-degrading enzymes BG and CBH while at the same time decreasing the activity of PO. Warming significantly reduced NAG activity but did not affect AlP activity. Furthermore, the warming treatment had a significantly lower ratio of N-degrading to P-degrading enzymes than the control [191].

Temperate forests had higher soil BG and NAG activities than subtropical and tropical forests, according to research along the North–South Transect in eastern China. Soil AP activities, on the other hand, showed an inverse trend, indicating that P deficiency limits the microbial nutrient demand in tropical forests. Soil BG and NAG activities were found to have significant negative correlations with MAT, MAP, soil C:P, and soil N:P ratios, but not with the soil C:N ratio [130]. Furthermore, research into ecological restoration efforts revealed increased biodiversity as well as C and N accumulation. Over an 11-year restoration period, the soil microbial biomass C and N increased with time, as did the activities of CAT, DHA, INV, Ure, and PO. Following that, these activities either remained consistently high or decreased [192].

Nowadays, all of the soil ecosystems are affected greatly by climate change consequences. This also changes the behavior of microbial enzymes and chemical cycles. Table 2 showcases the most representative research related to changes in soil enzyme activities in different soil ecosystems due to climate change.

The findings in Table 2 suggest that warming in alpine regions results in an increase in the activities of INV and AP. On the other hand, the activity of Ure differs in different ecosystems, showing either increases, declines, or no changes. The examination of the BG, AP, LAP, NAG, and CAT activities reveals that these enzymatic alterations are influenced by both global climate warming and vegetation seasonality. Notably, significant variations are observed, mainly in cold regions. A comprehensive database of all the soil enzyme activity studies would greatly improve the process and accuracy of interpreting novel findings and formulating long-term conclusions.

### 4.2. Strategies for C Sequestration and Enzymes Activities

Terrestrial soils are the biosphere’s main reservoirs of organic C. The mineralization of this organic matter by microbes has a significant impact on the global C and nutrient cycles, plant productivity, and atmospheric composition [20]. Soil C depletion can harm soil characteristics, such as compromised soil structure, decreased aggregate stability, decreased water retention capacity, restricted nutrient accessibility, and increased erosion potential [201]. According to the IPCC, CO_2_ concentrations will rise from 400 ppm to 1000 ppm by the end of the century [202]. Given that the soil CO_2_ concentrations are typically 10 to 15 times higher than the atmospheric levels, elevated atmospheric CO_2_ (eCO_2_) concentrations may have only minor effects on soil microbial communities in vegetated soil environments [174,203].

Pyrogenic organic C (biochar) utilization and land-use strategies are noteworthy approaches proposed for C sequestration, attracting significant research attention, especially in terms of their impact on soil enzymes. Biochar is made by pyrolyzing biomass waste and is used as a soil amendment or disposed of in landfills. Long-term application of biochar in tropical soils has the potential to improve soil fertility, especially in the creation of highly fertile soils known as Terra Preta or Amazonian Dark Earth [204]. Incorporating biochar is a novel strategy for increasing long-term soil C stocks while mitigating global warming by offsetting atmospheric C.

Land-use changes designed to increase natural C sequestration have the potential to capture and store significant amounts of CO_2_ each year. Conservation, management, and restoration of ecosystems, as well as C sequestration practices in agriculture, are all part of these changes. Sequestration practices in agriculture can improve the soil, air, and water quality, benefit wildlife, and also allow for increased food production [205,206].

#### 4.2.1. Land Use/Conversion

Research on the impact of land use on soil enzyme activity often produces contradictory results, calling into question the reliability of soil enzymes as consistent indicators of soil quality [207]. Although it is well established that soil use reduces the organic matter content, the effect on enzyme activity varies depending on the type of enzyme involved and the specific land use.

Land-use changes significant impact how global warming affects terrestrial ecosystems, impacting nutrient cycling, primary productivity, and biodiversity [123,208,209]. Climate change, together with land conversion, has a significant impact on soil microbial communities and their enzymes, which regulate the circulation of C and nutrients in terrestrial ecosystems [123]. Researchers discovered that switching from cropland to grassland increases C- and N-acquired enzyme activity while at the same time decreasing P-acquired enzyme activity [123]. This shift changes the enzyme stoichiometry ratios in grassland, suggesting changes in the C, N, and P limitations after land conversion. However, after cropland conversion, enzyme activities fluctuate over time [37]. Fungi also play an important role in mediating these changes during land conversion, influencing fluctuations in N- and P-acquired enzymes in particular [210]. The effect of climate warming on soil enzyme activities varies between cropland and grassland, indicating shifts in nutrient limitations as temperatures rise.

Several studies have also shown the importance of the soil pH, dissolved nutrients, and stoichiometry when it comes to influencing soil enzymes, particularly in grassland environments [123]. P limitation has been reported in both grasslands and temperate forest ecosystems [211,212]. Furthermore, during grassland restoration, microorganisms transition from P- to N-limitation, as demonstrated by changes in the soil extracellular enzyme stoichiometry [213]. A tropical watershed in Puerto Rico was studied to investigated enzyme activity via observing differences between soil orders [68]. Oxisols had higher enzyme activities than Inceptisols and Ultisols, which could be attributed to their higher organic matter content and finer texture [68].

Because of all the intricate relationships between the land use, soil enzyme activities, and soil quality indicators, it is necessary to conduct further research in order to uncover the consistent patterns and mechanisms guiding these interactions.

#### 4.2.2. Biochars

Assessing changes in the microbial community structure and enzyme activity can provide an understanding of the long-term effects of biochar on soil nutrient cycling processes [204]. Biochar has recently gained popularity due to its important role in adsorbing pollutants, improving soil fertility, and reducing greenhouse gas emission [214]. The effects of biochar on EEA and the contribution to C sequestration were analyzed to understand biochar’s impact on subtropical mangrove ecosystems. The results revealed that biochar treatments had varying effects on enzyme activity, with some enzymes increasing (PO, BG) and others decreasing (PP, NAG, AcP) [214]. However, only PP activity showed statistical significance. The observed increase in C sequestration could be attributed to a significant decrease in microbial abundance and enzyme activity as a result of the biochar intervention [214].

It was demonstrated that biochar application at ambient CO_2_ concentrations (aCO_2_) increased plant growth, which was further enhanced under eCO_2_ concentrations [215]. Biochar increased the activity of enzymes such as BG, Ure, and AlP under aCO_2_. However, only Ure activity increased with biochar addition under eCO_2_. Surprisingly, under eCO_2_, the positive effects of biochar on soil enzyme activities became less pronounced. The biochar types had varying effects on the bacterial diversity and fungal richness, particularly under aCO_2_ [215].

By summarizing various observations, researchers also discovered that biochar addition significantly altered soil C-degrading enzyme activities [216]. They found that soil ligninase activity targeting complex phenolic macromolecules increased by 7.1%, while cellulase activity degrading simpler polysaccharides decreased by 8.3%. These changes in enzyme activity were related to changes in soil C sequestration under various climatic, edaphic, and experimental conditions [216]. Short-term biochar addition (<1 year) significantly decreased cellulase activity while increasing soil organic C sequestration. On the other hand, long-term biochar addition (>1 year) increased ligninase activity, resulting in a smaller increase in soil organic C sequestration. These findings suggest that changes in enzyme activity over time, particularly an increased ligninase:cellulase ratio after biochar addition, may limit long-term soil C sequestration [216].

## 5. Influence of Intensive Agriculture on the Microbial Enzymatic Activity

Land-use intensification is a major anthropogenic factor of the 21st century, which has led to significant changes in local biodiversity and had a significant impact on ecosystem processes [1,217]. Over the last five decades, the global cultivated land area has increased by more than 500%, accompanied by a 700% increase in fertilizer use and a significant increase in pesticide application [218]. The choice and management of fertilizers have a significant impact on the soil microbiome, influencing various functions within agroecosystems [219].

A study evaluating distinct grassland sites with varying land-use intensity across two geographical regions in Germany found that enzyme activities were linked to abiotic soil properties irrespective of their geographical distribution [150]. Even though land-use intensity affects the spatial arrangement of enzymes, its effect on microbial biomass and EEA was found to be less significant than previously thought. Individual differences in location also played a role.

### 5.1. Influence of N and/or P Addition to Soil on the Activity of Soil Microbial Enzymes

Soil C sequestration has emerged as a viable strategy for improving soil quality [220]. It has been proposed that by increasing the SOC content, the soil physicochemical properties can be improved and thus increase crop yields. However, the global increase in atmospheric N deposition caused by agricultural and industrial activities has raised concerns. While N deposition has the potential to stimulate plant growth and increase soil C input, thereby increasing soil C storage, excessive N deposition has major consequences for the global C cycle and its interaction with climate change [221,222,223,224].

Because of the various effects on microbial enzymes, the impact of added N on microbial decomposition and soil C storage remains complex [225]. EEA may be altered through N addition by suppressing lignin-modifying enzymes (LMEs), which are responsible for breaking down resistant substrates, such as lignin, while at the same time raising cellulase activity [24,128,225,226,227,228]. Increases in soil C were also found to correlate with N-induced enzyme suppression [228]. Under N-limited conditions, microorganisms produce more LMEs because N-containing molecules are frequently protected by recalcitrant substrates, such as lignin. As a result, N additions may increase cellulase activity while decreasing LME activity [225,229] and influencing long-term soil C accumulation via a single enzyme system response to added N [225].

Studies on the impact of N addition on soil acidification and enzyme activity differ, with some indicating N-induced soil acidification and enzyme inhibition and others reporting stimulation or no effect [93,128,230,231,232,233]. Enzymes such as BG and AP are often used to assess changes in C and P cycling processes caused by environmental changes, with their activity influencing soil P conversion-related enzyme activity [93,227,234]. In particular, the results show that the addition of N inhibits BG and AcP [63]; however, the results can vary depending on the experiments.

The addition of N to the external environment may also increase inorganic N in the soil, which results in the formation of substances inaccessible to microorganisms and potentially reduces microbial activity. Hydrolytic enzymes (decomposing labile organic matter) and oxidative enzymes (breaking down resistant organic matter) are the two types of soil extracellular enzymes responsible for litter or organic matter degradation. Hydrolytic enzymes increase with N addition, while oxidative enzymes decrease [24,128,224,235]. Furthermore, long-term N addition can lead to soil acidification, which is detrimental to soil enzyme activity, resulting in decreased EEA [93].

Researchers investigated the effects of inorganic and organic amendments on the chemical properties of yellow clay soil, enzyme activities, microbial communities, and soil quality [220]. During the first three experimental seasons, they observed significant increases in the rice yields with fertilizer treatments. Adding organic matter had a greater impact on soil productivity. The NPKM (chemical fertilizer plus pig manure) treatment, especially, demonstrated the highest levels of nutrient availability, microbial biomass C, the greatest number of enzyme activities, and the microbial community. Because of their low PO, both NPKM and NPKS (chemical fertilizer plus straw) treatments may contribute to soil C sequestration. However, due to its high PP activity, the NPK treatment may limit SOC content, negatively affecting the labile organic C fraction [220]. The most relevant research related to N and P fertilizers and soil EEA is presented in Table 3.

The data compiled in Table 3 show that N and P inputs to various soil ecosystems have different effects on soil enzyme activity. The impact of N and P on enzymes like BG, AG, NAG, AcP, and Ure varies in different environments. These variations are influenced by the soil type, ecology, and climate. Long-term use of these fertilizers tends to alter the soil microbial dynamics, impacting enzyme activity significantly. For example, in some circumstances, N addition increases certain enzyme activities while inhibiting others. Similarly, P addition can reduce or increase particular enzyme activities, depending on the environment and soil qualities. This comprehensive information demonstrates the complexities of soil biochemical processes and emphasizes the importance of conducting context-specific assessments when evaluating the impact of fertilization on soil health and ecosystem functioning.

Recently, researchers have discovered a positive relationship between the activities of C-acquiring (BG) and N-acquiring (LAP, NAG) enzymes and changes in the C and N content of various human-managed grassland soils [25]. BG activity correlated positively with the soil C content, whereas LAP + NAG activity correlated positively with the soil N content. These relationships were found in a variety of grasslands, with varying soil pH and management history, but they were not found in intensively managed grasslands where high soil compaction negatively impacted enzyme activity. According to the authors, continuous nutrient fertilization increased the soil C content, resulting in a significant increase in BG activity compared to unfertilized soils [25]. Researchers discovered that different irrigation and N fertilization levels significantly affect soil health. Lower irrigation combined with higher N fertilization improved soil enzyme activities, nutrient content, and bacterial diversity more effectively than higher irrigation. Key soil enzymes like AcP and BG showed increased activity under these conditions [248]. The study’s findings highlighted that, while soil enzyme activities related to C and N acquisition are positively related to the soil C and N content, these activities are also noticeably responsive to changes in management practices such as the soil compaction and nutrient fertilization.

By conducting a 60-day incubation experiment using saline–alkaline soil and both types of N forms (organic/inorganic), scientists have found that in most cases, mixed N addition increased enzyme activities compared to single inorganic N addition [249].

Organic farming is becoming an increasingly relevant topic in the field of sustainable agriculture. The typical usage of agrochemicals, such as fertilizers and pesticides, undoubtedly has increased the agricultural yields. However, these materials have serious environmental consequences, compromising the soil, water, and air quality [250]. Organic farming offers a viable alternative for eco-functional development. This strategy focuses on using and improving natural resources and processes in order to maintain the soil–plant system’s natural balance. Organic farming aims to produce high-quality, healthy food while minimizing the presence of harmful residues and toxic substances, which benefits both humans as well as flora and fauna animals [251,252].

Effective field techniques in organic farming not only ensure consistent and high agricultural yields but also contribute to the environmental sustainability [253]. Composted animal manure (e.g., poultry, pig, cow) and green manure, often mixed with straw, are commonly recommended fertilizers in organic practices [252,254,255]. A thorough meta-analysis of the literature comparing organic and conventional cropping systems found that organic systems had significantly higher levels of microbial biomass, C, N, total phospholipid fatty acids, and enzyme activities such as DHA, Ure, and protease [251]. Despite these advantages, organic farming frequently results in lower crop yields than traditional approaches [252].

Further research in Bogor and Tasikmalaya Regencies revealed that soil enzyme activities, such as DHA and cellulase, were higher in organically cultivated soils than in conventionally farmed ones. However, soil Ure activity in organic farming was decreased compared to conventional farming [250]. These soil enzymes were found to be significantly correlated with critical soil parameters, such as the organic C, total N, accessible P, and K^+^, lending support to the claim that organic farming promotes soil fertility and plant productivity. However, it was noted that a minor amount of Ure fertilizer may still be required [250].

In a three-year study of twenty-four greenhouse-grown vegetable crops, the effects of different compost application rates were compared to chemical fertilizer and no-fertilizer treatments. The study discovered that compost treatments dramatically boosted soil enzyme activity, including DHA, cellulase, protease, and Ure [256]. It was found that organic amendments increased enzyme activity by about 50–75% [257]. Additionally, researchers discovered that both compost and manure boosted soil enzyme activity, with manure-treated soils having a higher risk of N loss due to increased nitrification and denitrification potentials [258].

Treatments with straw mulching and organic fertilizer increased soil enzyme activity, particularly cellulase, during various wheat growth stages [253]. Two organic farming systems in a tropical climate were evaluated: one with green manure and bokashi, and another with composted poultry manure. Both systems, characterized by low N inputs, exhibited positive relationships between pH, Ca, P, AlP, and BG activity and organic treatments [252]. Along a similar line, it was suggested that employing sheep farming leftovers as organic fertilizer results in enhanced soil health and greater barley yield [259].

Lastly, incorporating maize residues into soil has been recognized as an effective strategy for minimizing chemical N fertilization while at the same time maintaining yield and soil fertility [260]. These examples show that organic farming considerably increases soil enzyme activity, providing a long-term strategy for improving soil fertility and environmental health.

The combined effect of warming and N or P addition is another interesting research topic. It was found that warming increased AlP activity by 35.8% but inhibited cellulase activity by 30% in a study investigating the response of soil enzyme activity to N addition and experimental warming [233], whereas N addition only increased Ure activity by 34.5% and AP activity by 33.5% without affecting cellulase activity. Furthermore, cellulase and AP activities were strongly related to the soil temperature and water content, whereas Ure activity was primarily related to the soil N availability. According to the study, climate change not only has a significant impact on EEA but also on soil nutrient mineralization processes [233].

Several studies have also shown that P limitation is a global phenomenon that affects many terrestrial ecosystems rather than being limited to a few [230,261,262]. For example, a decrease in the soil bioavailable P concentration was found as the MAT increased in 80 grasslands across China [263]. Meta-analyses on bioavailable P were conducted in global natural (seminatural) soils and found negative correlations between MAT and MAP and the soil bioavailable P concentration [264]. Furthermore, the parent material, sand content, pH, organic C, and Al-Fe oxide content have been identified as important factors influencing soil P bioavailability on a global scale [230,265]. According to reports, soil microbial biomass C and AcP are important predictors of soil P bioavailability in agro- and natural ecosystems, though they appear to be less influential than the total soil P [266]. Enzymes such as PHY have also been identified as critical components of the global P cycle [266].

Another investigation was conducted into the effects of P and N fertilization on the S dynamics in soils from two tropical forest plantations [267]. P fertilization was found to have a significant effect on the availability of S in soil, as phosphate has a greater tendency to bind to mineral soil than sulfate. This phosphate fertilization characteristic may desorb sulfate, resulting in a decrease in the available soil S [267]. Additionally, the study’s findings revealed a significant decrease in the soil exchangeable S in P-fertilized plots. In these P-fertilized plots, however, ARS activity increased slightly. The ratios of soil ARS activity to C- and/or N-acquiring enzyme activities also had a tendency to increase. This pattern could suggest that P fertilization exacerbated S deficiencies by increasing the microbial demand for S relative to C and N. N addition, on the other hand, resulted in a significant decrease in soil ARS activity and altered enzyme activity ratios but had no effect on the exchangeable S. The researchers concluded that ARS may be more sensitive to N fertilization than to the soil pH, emphasizing the importance of careful P fertilization management in tropical forest plantations to mitigate its negative effects on soil S availability [267].

### 5.2. Influence of Herbicides and Other Agriculture Additivities on the Soil Enzymes

Pesticides play an important role in preserving agricultural produce quality by controlling plant pathogens. Their use, however, can have an impact on the soil microbial community and alter its biochemical activities, which results in changes in soil enzyme functionalities [155]. In one study, the effects of 20 commercial pesticides on BG, CBH, and BX in 3 distinct south Australian agricultural soils were assessed. Pesticides stimulated cellulolytic and chitinolytic activities in soils, according to the findings [155].

Specifically, the insecticide cypermethrin has been shown in multiple studies to increase soil bacterial populations and enhance cellulase activities [268,269]. An investigation explored the impact of the herbicide atrazine on soil enzyme activities and discovered its widespread use in Chinese agricultural production as well as the widespread environmental concerns associated with it [270]. Non-target soil microorganisms are frequently affected by atrazine, an endosynthetic herbicide used before and after selective seeding to inhibit the growth of the target plants [271,272]. Atrazine application also had a significant impact on soil Ure and cellulase activity but had no effect on saccharase activity. With atrazine application, the levels of Ure and cellulase in the soil rapidly decreased, indicating an inhibitory effect on Ure [270]. These findings imply that atrazine can influence N conversion in the soil. Because soil Ure activity is positively associated with soil fertility, atrazine application may reduce soil fertility levels [273].

Mesotrione is a selective triketone herbicide that has been used in corn production over the past 15 years [274]. It was studied with a focus on its effects on soil enzyme activity. The study discovered that the activities of Ure and acid AcP remained generally stable, with no major differences between the mesotrione-treated and control groups at the conclusion of the exposure period. The activity of BG decreased in soils treated with 5.0 mg/kg of mesotrione [274]. Furthermore, in a separate study of sandy loam soil, it was discovered that when a 50 mg/kg mesotrione concentration increased the soil microbial biomass, it also decreased soil DHA activity [275]. These findings show that mesotrione’s effects on soil enzymes and microbial biomass vary depending on its concentration and the enzyme or microbial process under investigation.

While fungicides successfully eliminate fungal diseases in crop protection, leakage into other environmental components might have severe and irreversible consequences [276]. According to research, azoxystrobin inhibits the action of DHA, CAT, Ure, AcP, and AlP. However, DHA was the most resistant to the fungicide’s effects, while AlP recovered the fastest in the soil [276]. Pesticides can have an immediate effect on enzymes via reducing catalytic activity or altering microbial activity [277]. Pesticides are particularly toxic to DHA, BG, and AlP, according to research [276].

Biodegradable plastics, particularly as a replacement for agricultural mulch, have been gaining attention in recent years [278]. Studies on polylactic acid microplastics (PLA MPs) have shown that excessive concentrations can have a negative impact on the soil characteristics, soil microbiology, and short-term plant development [278]. PLA MPs have been associated with a lowered soil pH, increased redox potential, and increased fungal and bacterial abundance [279]. In contrast, studies on conventional MPs show that polypropylene promotes fluorescein diacetate hydrolase (FDAse) activity in soil [280], whereas polyethylene (PE) and polyvinyl chloride (PVC) reduce FDAse activity while increasing Ure and AcP activity [281]. Research has shown that a 10% PLA concentration enhanced Ure and AlAP activity while inhibiting FDAse activity [282]. Similarly, it was found that degradable MPs, such as PLA, increased Ure, AP, and CAT activity, with higher enzyme activity levels at higher concentrations (2%) compared to lower concentrations (0.2%) [283]. PLA MPs could potentially enhance microbial activity by increasing C bioavailability in the soil, resulting in increased enzymatic activity. Furthermore, the C:N imbalance caused by PLA MPs may drive plant competition for available nutrients with soil microorganisms, hence increasing enzyme production [278].

## 6. Current Challenges and Future Perspectives

Soil fertility is an important component of our ecosystem, which supports agricultural output and environmental sustainability [284]. However, it faces major threats from human-induced issues like desertification, biodiversity loss, and nutrient depletion. The complexity of soil ecosystems, which are influenced by a variety of elements, such as climate, geography, and human activities, makes assessing and maintaining soil fertility a difficult task [285].

The heterogeneity and unpredictability of soil biochemical characteristics as markers of soil health and fertility complicate the task even further. Field experiments, which are frequently influenced by external factors, produce a wide range of outcomes that can be difficult to generalize [286]. Similarly, controlled laboratory research may not fully reflect the complicated interactions found in natural soil ecosystems [287,288]. Despite these obstacles, soil microbial enzymes have been identified as potentially useful instruments for monitoring environmental changes and assessing soil health. Their reactions to various treatments provide important information about the soil’s state and functionality [5].

One of the primary issues in this field is the variability of soil enzyme reactions to climate changes across habitats. This variation can be attributed to changes in the soil composition, vegetation, and local environmental conditions. Creating a global database that gathers information on changes in soil microbiota and enzyme activities as a result of climate change would be extremely beneficial [289]. A database like this would allow for a more thorough understanding, more accurate predictions, and more effective management tactics. Other issues that require additional research include the influence of biochar application and the challenges created by rising CO_2_ levels [290]. Additionally, the impact of biochar and high CO_2_ levels on soil microbial populations and enzyme activity is not well understood and requires further research [215]. Furthermore, to improve our understanding of soil ecosystem processes, the interaction between soil microbial EEA, soil organic C, and nutrient dynamics must be incorporated into global biogeochemical models [25,215].

Urban agricultural soils present a unique set of challenges. These soils are exposed to intense human activity, such as urbanization and industrialization, which affects their composition and functioning significantly. Therefore, it is important to develop sensitive and reliable indicators for assessing the conditions of urban agricultural soils in these situations [291,292].

It is critical to understand the causes of soil deterioration to develop efficient restoration solutions. Natural processes like erosion and disasters, and manmade activities including as deforestation, agriculture, and urbanization, can all cause soil damage. Resolving these issues fully is critical to long-term soil management [293]. It is critical to integrate findings from controlled-manipulation studies that explore the effects of global change drivers on soil ecosystems. These studies shed light on how plants and soils may react to factors such as N deposition, warming, and rising CO_2_ levels [294]. A thorough examination of these consequences, considering both individual and cumulative effects, would improve our understanding of soil fertility in the context of global change.

Long-term, ecosystem-scale studies spanning diverse locations should be prioritized in future research to better understand the multifactor effects of global change drivers on soil health and fertility. Such research will be helpful in the development of agricultural strategies for ecological intensification and thus contribute greatly to sustainable soil management and overall environmental health.

## 7. Conclusions

This article showcases the significance of enzymes produced by soil microbiota, such, as α-glucosidases and β-glucosidases, phosphatases, ureases, N-acetyl-glucosaminidases, peptidases and others. It emphasizes how these enzymes play a role in maintaining soil health and promoting C sequestration. They assist in breaking down matter and facilitating N cycling, which are essential for preserving soil fertility. The research conducted on enzyme activity under different conditions, like various soil temperature, moisture levels, pH balance and climate zones, has revealed their susceptibility to climatic and edaphic factors. Moreover, the article also sheds light on the potential of these enzymes as indicators of soil health and fertility. The impact of climate change and intensive agriculture on enzyme activity has been thoroughly studied as well, highlighting the role played by enzymes in the C cycles. The report encourages more research in order to gain an understanding of these enzymes and develop effective strategies to utilize their potential for mitigating the effects of climate change through efficient approaches to C sequestration.

By emphasizing the relationship between enzymatic activity in soil and broader environmental processes, this review underscores the importance of preserving soil health not only for agricultural productivity but also for ecological sustainability. As we move forward, it becomes more and more crucial to connect our knowledge of soil microbiology with climate science to develop efficient approaches for management and conservation.

## Figures and Tables

**Table 1:** Microbial enzyme activity in different soil ecosystems. Acid phosphatase (AcP), alkaline phosphatase (AlP), arylsulphatase (ARS), β-1,4-glucosidase (BG), β-1,4-N-acetyl-glucosaminidase (NAG), β-xylosidase (BX), catalases (CAT), cellobiohydrolase (CBH), dehydrogenases (DHA), invertase (INV), leucine aminopeptidase (LAP), mean annual temperature (MAT), peroxidases (PP), phenol oxidases (PO), phytases (PHY), soil organic carbon (SOC), soil organic matter (SOM), ureases (Ure).

Soil Ecosystem/Location	Enzyme Activities and Important Remarks	Ref.
China’s forest ecosystems	The activity of CAT, PO, AcP, AlP, and proteases varied significantly across forest types. In particular, primosols, cambisols, and argosols have higher CAT and Ure activity than ferrosols. Enzyme activities decreased with increasing soil depth but increased with SOM. Both PO and Ure had a negative connection with MAT, whereas CAT, INV, and protease activities showed a complex pattern: they reduced at temperatures below 2.5 °C, increased between 2.5 °C and 17.5 °C, and then fell again at temperatures over 17.5 °C. Protease activity was somewhat positively correlated with MAP, but CAT, PO, and Ure activities were negatively correlated. The activity of CAT, INV, AcP, AlP, Ure, and proteases increased and then decreased with altitude.	[127]
Two afforested lands (coniferous woodland and leguminous shrubland), Wulongchi Research Station, Hubei Province, China	The C:N ratio of enzymes in afforested areas was much greater than in open areas. This ratio was found to be lower in forests compared to shrublands.	[132]
Two forests, *Betula albosinensis* (Ba) and *Picea asperata* Mast. (Pa); Qinling Mountains, China	The average seasonal enzyme activities of BG and BX in Ba forest soils were 30.0% and 32.3% greater, respectively, than those in Pa soils, whereas CBH activity was 19.7% lower. Local organic C in the soil had a substantial positive connection with CBH, BG, and BX enzyme activity. Pa soil had a lower SOC content and lower BG and BX enzyme activity than Ba soil. This was largely owing to differences in litterfall and root exudates between Pa and Ba. During the summer and autumn seasons, CBH, BG, and BX enzyme activity increased in both Pa and Ba forest soils.	[148]
Different soil depths in subtropical forests; soil layers (0–10, 10–20, 20–40, 40–60 cm) in a natural secondary evergreen broad-leaved forest and a Chinese fir (*Cunninghamia lanceolata*) plantation forest in subtropical China	Microbial C and P limitation changed with soil depth, with microorganisms in soil below 20 cm in both forests requiring greater N. The activity of C-acquiring (BX + CBH + BG), N-acquiring (NAG + LAP), and P-acquiring enzymes decreased as soil depth increased. However, the regularity of enzyme activity across the soil profile indicates an imbalance in microbial nutrient demand at different soil depths.	[149]
The 18 independent grassland sites differing in their land-use intensity in two geographic regions: the Hainich National Park in the middle of Germany and the Swabian Alb in south-west Germany	Enzyme activities related to C-acquiring and N-acquiring (BG, BX, and chitinase), as well as organic C, total N, extractable organic C, and mineral N, were found to be higher in the Swabian Alb (Leptosols) than in the Hainich National Park (mostly Stagnosols). Bulk density was found to be negatively correlated with microbial biomass, Ure activity, organic C, and total N. The activities of BG, chitinase, BX, AP, and Ure were impacted by local abiotic soil characteristics but showed little geographical association.	[150]
Regional-scale karst area, southwest China; secondary forest, shrubland, grassland and cropland underlain by either dolomite or limestone	The activity patterns of extracellular enzymes involved in C, N, and P cycling varied significantly between dolomite and limestone, as well as across the four types of land use. These variations in enzyme activity were impacted by changes in land use.	[151]
The collapsing, collapsed, and an unaffected site of a thermokarst feature on the Northern Qinghai–Tibetan Plateau	In the top 0–20 cm layer, collapsing soils had significantly lower INV activity than control and collapsed soils. At a soil depth of 0–10 cm, collapsing soils had the highest CAT activities and the lowest Ure activities among the three circumstances. Light fraction C content, C:N ratios, and moisture content emerged as important indicators of enzyme activity. Among the six enzyme activities measured, four showed significant differences in the upper 10 cm of soil.	[152]
Permafrost regions of the middle and western Qinghai–Tibetan Plateau; cold, arid steppe, with an active layer thicker than 2 m	The activities of enzymes such as INV, CAT, amylase, cellulase, Ure, and AlAP were measured. Soil enzyme activity was observed to be higher in Stipa roborowskyi Roshev vegetation communities than in Carex moorcroftii Falconer ex Boott communities. The alpine cold desert had the lowest soil enzyme activity.	[119]
Tundra soils, which contain low concentrations of soil nutrients, low pH, store a large proportion of the global soil C pool	The potential activity of BG was discovered to rise with increasing nutritional levels. In contrast, as soil pH increased, BG activity decreased. When nutritional restrictions were corrected through fertilization, microbial biomass and enzymatic capacity for cellulose decomposition increased, presumably improving SOM decomposition. However, increasing soil pH was found to reduce the enzymatic capacity for cellulose degradation, presumably due to changes in the bioavailability of organic substrates.	[153]

**Table 2:** Microbial enzyme activity in soil due to climate changes at different soil ecosystems. Alkaline phosphatase (AlP), arylsulphatase (ARS), β-1,4-glucosidase (BG), β-1,4-N-acetyl-glucosaminidase (NAG), β-xylosidase (BX), catalases (CAT), cellobiohydrolase (CBH), dehydrogenases (DHA), extracellular enzyme activity (EEA), invertase (INV), leucine aminopeptidase (LAP), mean annual temperature (MAT), peroxidases (PP), phenol oxidases (PO), phytases (PHY), soil moisture (SM), soil organic carbon (SOC), soil organic matter (SOM), ureases (Ure).

Soil Ecosystem/Location/Climate Zone	Effect on Soil Enzyme Activity and Soil	Ref.
**Alpine**		
Alpine meadow, northwestern Sichuan, China	At a soil depth of 0–10 cm, experimental warming enhanced AcP, INV, and Ure activities, as well as accessible nutrients, while lowering CAT activity and SOM levels. Warming at 10–20 cm deep enhanced CAT activity, SOM, accessible N, and K^+^ while decreasing INV activity.	[124]
Alpine swamp meadow, Qinghai–Tibetan Plateau, alpine grasslands, permafrost regions, a 3-year experiment with two warming levels (2.7 °C and 5.3 °C)	NO_3_^−^N and SM were critical in explaining large differences in soil enzyme activity. Warming increased INV and amylase activity throughout the growing season while decreasing Ure activity, but had no significant influence on CAT or cellulase activity.	[193]
3-year in situ soil core incubation experiment, a 2431-m altitudinal gradient in an alpine-gorge region, the eastern Qinghai–Tibet Plateau	Between 2013 and 2017, subalpine coniferous forests and alpine meadows had higher INV, Ure, and AcP activities than dry valley shrubland and valley-mountain ecotone forests. EEA’s sensitivity to seasons reduced with altitude.	[194]
Old-adapted alpine grassland of the Tibetan Plateau	Enzymes that degrade low-quality polymers remained temperature sensitive above 25 °C. Several enzymes’ substrate affinity rose up to 20 °C, but their K_m_ increased rapidly at 25 °C, lowering catalytic effectiveness.	[195]
Alpine meadow, alpine steppe and cultivated grassland, Qinghai–Tibetan plateau; 3-year warming, enhanced precipitation and yak overgrazing	Despite various treatments being applied, the activity levels of sucrose and AP remained consistent. In contrast, overgrazing in cultivated grasslands led to an increase in Ure activity and microbial biomass (N).	[196]
**Grasslands**		
Temperate grasslands of northern China during the growing season of 2013	Temperate grasslands had lower enzyme C:N and C:P ratios than tropical soils. The enzyme ratios changed with soil depth, and log-transformed enzyme ratios differed from global ratios, indicating a greater investment in N-acquiring enzymes in temperate grasslands.	[137]
Temperate grassland of northern China at two depths of 0–10 and 10–20 cm	Due to warming AcP activity increased at 0–10 cm depth, as did NAG at 10–20 cm depth, whereas BG and AcP activity declined in the subsurface. Increased precipitation boosted NAG, LAP, and AlP activity in both surface and deep soils.	[174]
**Forest**		
Forest soils from the fragile cold ecosystems, Western Patagonia, Chile	NAG activity, like microbiological activity, was more temperature sensitive than BG. Soil total nutrients had a greater influence on enzyme K_cat_ than accessible nutrients throughout vegetation succession, with BG being more sensitive to severe temperatures.	[197]
Ziwuling forest region of the Loess Plateau	During extended vegetation succession, total soil nutrients had a greater impact on enzyme K_cat_ than accessible nutrients. The kinetic characteristics of soil enzymes changed dramatically over this succession. BG was more responsive to severe temperatures than NAG or AlP. At both low (5 °C) and high (35 °C) temperatures, the V_max_, half-saturation constant (K_m_), and other kinetic parameters of BG were disconnected.	[198]
**Other**		
Permafrost peatland near the Tuqiang Forestry Bureau in the Great Xing’an Mountain, Heilongjiang Province, northeast China	PO demonstrated a greater response to temperature fluctuations compared to enzymes like BG, NAG, and AcP. The combined effects of rising temperatures and water flooding resulted in a synergistic impact, leading to an increase in both bacterial and fungal populations, as well as the activity levels of various soil enzymes.	[199]
High Arctic dry tundra, continuous permafrost zone, Cambridge Bay, Nunavut, Canada	The activities of BG, cellobiase, NAG, LAP, and PO peaked in June and dropped throughout the summer. Environmental conditions have a major impact on hydrolase activity fluctuations, influencing EEA and the structure of the Arctic microbial community.	[176]
Mediterranean climate gradient in southern California	V_max_ of most enzymes was more sensitive to temperature in cooler environments, particularly during the dry season. K_m_ was more sensitive in warmer areas, indicating enzyme build up in drier regions, which influenced respiration following rewetting occurrences.	[181]
Karst region of southwestern China	As vegetation succession progressed, AlP activity increased and Ure dropped. Ure was positively connected with rock outcrop cover but negatively with litter N, soil accessible N, and pH, whereas AlP showed the opposite correlation.	[200]

**Table 3:** Influence of N and P fertilization on the soil enzyme activities. α-glucosidase (AG), acid phosphatase (AcP), alkaline phosphatase (AlP), aryl-sulfatase (ARS), β-1,4-glucosidase (BG), β-1,4-N-acetyl-glucosaminidase (NAG), β-xylosidase (BX), cellobiohy-drolase (CBH), chemical fertilizer of N (NPK), dehydrogenases (DHA), extracellular enzyme activity (EEA), invertase (INV), leucine aminopeptidase (LAP), phenol oxidases (PO), peroxidases (PP), soil moisture (SM), soil organic carbon (SOC), ureases (Ure).

Soil Ecosystem/Location and Used Fertilizers (If Used)	Enzyme Activities and Other Important Remarks	Ref.
**N addition**		
Soils from hardwood forests at Bear Brook, Maine, and Fernow Forest, West Virginia.	V_max_ and K_m_ for AG, BG, BX, CBH, and NAG increased with N addition, especially at Fernow. N fertilization reduced Km at Bear Brook, but had varied effects at Fernow. Both V_max_ and K_m_ were temperature sensitive, with BX demonstrating a substantial relationship between N and temperature for K_m_ in hardwood forest soils.	[7]
Agricultural field, yellow clayey soil, located in Jingshan county, Hubei, China. NPK, NPK plus green manure (NPKG), NPK plus pig manure (NPKM), and NPK plus straw (NPKS) were used for fertilization.	NPKM treatment increased ARS, BG, AG, NAG, and CBH activities compared to the unfertilized control. Except for phosphomonoesterase and NAG, the NPKG and NPKS treatments had equal or lower activity levels. Low PO activity may result in soluble phenolic build-up, which inhibits hydrolytic enzymes.	[220]
The effect of simulated N deposition in six forest ecosystems in eastern China. Soil samples from three blocks × four soil depths (0–10 cm, 10–20 cm, 20–40 cm and 40–60 cm) were collected.	Four to five years of N addition exhibited little effect on BG, CBH, PO, PP, NAG, LAP, and AcP activities and ratios, with very minimal site-specific responses for AcP.	[236]
Soil of a Korean pine plantation in which different concentrations (0, 20, 40, 80 kg N ha^−1^ year^−1^) of ammonium nitrate were applied for 5 consecutive years.	Moderate N addition (40 kg N ha^−1^ year^−1^) significantly reduced Ure activity, with all three treatments exhibiting lower protease activity than control. There was no connection discovered between microbial community structure and four mineralizing enzymes, and N concentrations had no effect on soil pH.	[237]
Nash’s Field long-term grassland experiment established on acidic soils at Silwood Park, Berkshire, UK. 19 years of chronic N-only addition to permanent grassland was tested.	Chronic N addition over 19 years improved C storage and BG activity in thick soils. N fertilizer decreased root C:N ratios, which increased microbial demand for root C. Lime application reduced BG activity and root mass in high-pH soils.	[228]
16-year experiment conducted in a typical grassland in northern China.	N addition inhibited BG and AcP, while H_2_O addition had no effect on BG but lowered AcP. Soil enzyme activity was mostly affected by soil microbial biomass C.	[93]
Short-term N addition (NH_4_NO_3_) in a sandy grassland and semi-fixed sandy land in the Horqin Sandy Land, northern China.	NAG activity and soil microbial features remained constant across N levels and locales. N addition increased BG activity in sandy grassland and semi-fixed sandy land.	[223]
Semi-arid grassland in China	N addition increased C-acquiring enzyme activity but lowered N-acquiring enzyme activity in low-precipitation years, while it stimulated all enzymes in high-precipitation years.	[238]
Temperate and alpine grassland ecosystems in China	Although N and P additions had little effect on SOC concentration, they did change soil pH, total N, and total P content. Only AcP was inhibited by P addition at the temperate meadow site; other EEA and stoichiometric ratios were unaffected.	[239]
Typical meadow soil (Vertisols) near to Görbeháza, Debrecen, Hungary. The field is cultivated by a rain-fed maize monoculture and fertilized continuously at different doses of NPK	Long-term NPK fertilization improved microbial tolerance to fluctuations in SM content. High rainfall decreased soil NO_3_^−^ and nitrification rates. The EEA responded more to SM than NPK, with the highest AP, DHA, and INV activity in the drier year and the highest Ure activity in the wettest year. High NPK rates lowered soil DHA activity.	[240]
Five-year field fertilization experiment to study how N addition affected soil enzyme activity patterns in the topsoil (0–20 cm) and subsoil (20–40 cm) in a Tibetan alpine meadow	N addition altered soil EEA via pH variations. At greater N rates, N-induced soil acidification enhanced BG and Ure activities while maintaining AcP and decreasing PO activity.	[241]
Typical steppe ecosystem in Inner Mongolia	N additions reduced soil N-related hydrolytic enzyme activity.	[242]
**P addition**		
Subtropical/tropical moist forest in Dinghushan Biosphere Reserve (DHSBR) which is an UNESCO/MAB site located in the middle Guangdong Province in southern China	P addition reduced AcP activity while increasing LAP activity but had no effect on LAP specific activity and lowered NAG specific activity. CBH, AG, and BG exhibited no significant reaction, however P addition reduced BX activity. It also lowered PO and PP activity, indicating a decrease in microbial enzyme synthesis in P-poor tropical forests.	[243]
Mesophytic deciduous forest soil on the unglaciated portion of the Allegheny Plateau, southeast Ohio, USA	All treatments lowered extracellular AP activity across both soil horizons. The reduction in AP resulted in a relative increase in C acquisition compared to N and P acquiring enzymes, affecting overall ecoenzymatic stoichiometry.	[244]
Desert steppe in Eastern Yanchi County, Ningxia Hui Autonomous Region, Northwest China	The enzyme stoichiometry was 1.2:1:1.5. Soil BG activity declined with heat and P addition, while AIP was reduced by warming, P addition, and warming combined with P addition.	[245]
Topsoil (0–75 mm) from a grazed pasture receiving contrasting P inputs. The field study was situated at Winchmore, New Zealand	Long-term P input decreased AcP while increasing AlP activity, which peaked in the summer and dropped in the winter. AcP and AlP linked positively with soil temperature but negatively with SM.	[246]
Two cotton cultivars and three phosphorus (P) levels. A pot experiment was conducted in 2017 at the Baibi station, Anyang, Henan, China	The activities of INV, cellulase, and urea in cotton soil decreased significantly after P addition.	[247]

## Data Availability

Data sharing is not applicable to this article.

## Abbreviations

AG—α-glucosidase, AP—alkaline/acid phosphatase, AcP—acid phosphatase, AlP—alkaline phosphatase, ARS—arylsulfatases, BG—β-1,4-glucosidase, BX—β-xylosidase, CAT—catalases, CBH—cellobiohydrolase, Ea—activation energy, EEA—extracellular enzyme activities, EST—carboxylesterases, DHA—dehydrogenases, DNRA—dissimilatory nitrate reduction to ammonia, INV—invertase(s), LAP—leucine aminopeptidase, LIMEs—lignin-modifying enzymes, LIP—lipolytic enzymes/lipases, LPMO—lytic polysaccharide monooxygenase, MAT—mean annual temperature, MAP—mean annual precipitation, MICP—microbially induced carbonate precipitation, MPs—microplastics, NAG—β-1,4-N-acetyl-glucosaminidase, NR—nitrate reductase(s), PAEs—phosphate esters, PHY—phytases, PLA—polylactic acid, PO—phenol oxidases, PP—peroxidases, ROS—reactive oxygen species, SOC—soil organic carbon, SM—soil moisture, SOM—soil organic matter, Ure—ureases, XYL—xylanase.
